# Network analysis of Iranian's health insurance ecosystem before and after the introduction of Universal Health Insurance law

**DOI:** 10.1186/s41256-023-00302-5

**Published:** 2023-05-22

**Authors:** Rohaneh Rahimisadegh, Somayeh Noori Hekmat, Mohammad Hossein Mehrolhassani, Mohammad Jafari Sirizi

**Affiliations:** 1 Health Services Management Research Center, Institute for Futures Studies in Health, Kerman University of Medical Sciences, Kerman, Iran; 2 Health Foresight and Innovation Research Center, Institute for Futures Studies in Health, Kerman University of Medical Sciences, Kerman, Iran; 3 Department of Health Management, Policy and Economics, Faculty of Management and Medical Information Sciences, Kerman University of Medical Sciences, Kerman, Iran; 4 National Center for Health Insurance Research, Tehran, Iran

**Keywords:** Health insurance, Ecosystem, Actors, Network analysis, Universal health insurance law, Iran

## Abstract

**Introduction:**

The policy-making process in health reform is challenging due to the complexity of organizations, overlapping roles, and diversity of responsibilities. The present study aims to investigate and analyze the network of actors in the Iran health insurance ecosystem regarding the laws before and after the adoption of the Universal Health Insurance (UHI).

**Methods:**

The present study was done by sequential exploratory mixed method research, consisting of two distinct phases. During the qualitative phase, the actors and issues pertaining to the laws of the Iranian health insurance ecosystem from 1971 to 2021 were identified through a systematic search of the laws and regulations section of the Research Center of the Islamic Legislative Assembly website. Qualitative data was analyzed in three steps using directed content analysis. During the quantitative phase, in order to draw the communication network of the actors in Iran's health insurance ecosystem, the data related to the nodes and links of the networks was collected. The communication networks were drawn using Gephi software and the micro- and macro-indicators of network were calculated and analyzed.

**Results:**

There were 245 laws and 510 articles identified in the field of health insurance in Iran from 1971 to 2021. Most of the legal comments were on financial matters and credit allocation, and the payment of premiums. The number of actors before and after the enactment of the UHI Law was 33 and 137, respectively. The Ministry of Health and Medical Education and the Iran Health Insurance Organization were found the two main actors in the network before and after the approval of this law.

**Conclusions:**

Adopting a UHI Law and delegating various legal missions and tasks, often with support to the health insurance organization, have facilitated the achievement of the law objectives. However, it has created a poor governance system and a network of actors with low coherence. Based on the results of the study, it is suggested to reduce actor roles and separate them for better governance and to prevent corruption in health insurance ecosystem. Introducing knowledge and technology brokers can be effective in strengthening governance and filling the structural gaps between actors.

## Introduction

Health is pivotal to improving the quality of life for people in all countries [1]. The World Health Organization (WHO) believes health systems should maintain and promote individuals' health while meeting their expectations and protecting them from disease and financial burdens [2]. Increasing financial protection and improving access to health services form the core of a financing and insurance system [3]. Health insurance supports healthcare system and acts as an intermediary organization between patients and providers [4]. Their functions include revenue collection, accumulation, and service purchasing, providing financial protection and promoting higher financial gains for the community's health and well-being [2, 5].

Health insurance functions aid policymakers in health system reforms as a key topic [6]. Systemic problems, failure in achieving results, changing disease patterns, demographic changes, the introduction of new equipment and technologies, increased health system costs, and on the other hand, the lack of resources are factors that necessitate and accelerate reforms in the health system [7]. Health system reforms aim to improve quality, efficiency, and cost by enhancing the financing system [8]. Healthcare and insurance system reforms need cooperation from different government and non-government entities, which is a major challenge in many countries, including Iran [9]. Reforming healthcare is challenging due to stakeholder opposition, as implementing policy changes affects their interests [8, 10].

Reforming policies in Iran's mixed health system [11] is challenging due to organizational complexity, multiple governance institutions and decision-making bodies, and inefficiency. To address this, fundamental actors, roles, and interactions must be identified [12, 13]. Now, Formation and implementation of policies involve multiple actors in a complex, competitive network [14]. Therefore, having an analytical tool is crucial for policy-makers to simplify policy networks and promote implementation [15]. Social network analysis aids in identifying effective organizations and key links [16]. Social network analysis visualizes power maps and explains social phenomena by analyzing the structural and communicational features of related actors. It's used in social sciences, policy-making, and management to describe actor power and influence in policies and laws [15, 17]. In political literature, networks solve complex organizational problems [18].

After the Iran–Iraq War ended in 1988, Iran's economic reconstruction raised health service costs. The Universal Health Insurance Law was drafted in 1992 to address this. UHI Law of 1994 aimed to cover 60% of Iran's uninsured population. MSIO was created in October of that year to cover a broad population within 5 years [19]. Before the UHI law, there was no clear legislation on insurance companies and only service coverage was mentioned. The enactment of the UHI Law and the subsequent establishment of the MSIO resulted in desirable cohesion and consistency between the policies and laws in health insurance in Iran [20]. After UHI Law approval, MSIO was formed, followed by the High Council of Health Insurance (HCHI) in the Ministry of Health and Medical Education (MOHME). HCHI aims to expand insurance coverage, set policies, decide on packages, rates and tariffs, monitor performance, and evaluate the system to ensure that all insurance plans follow the same laws and regulations [21]. The UHI Law aimed to provide health insurance coverage for all eligible individuals within 5 years, with a focus on uninsured populations in urban areas [22]. It increased population coverage and financial protection against health costs through various insurance schemes [23].

In Iran, the financing of the health system is a combined budgeting system and is mainly based on social insurance. Health insurance is funded from four main sources of tax revenue, petroleum sale revenue, out-of-pocket expenditure, and premium contributions [24]. The Social Security Organization (SSO) and IHIO are the two main purchasers of health services in the country [25]. The Health Insurance Organization was initially named the MSIO and operated under the Ministry of Cooperative Labor and Social Welfare (MCLSW) like the SSO. It was later transferred to the MOHME in 2017 [26]. In Iran, organizations that provide health insurance services (insurers) are divided into three main groups based on their functional nature: Social Health Insurance (SHI) IHIO, SSO, and Armed Forces Medical Services Insurance Organization (AFMSIO), Institutional Health Insurance Funds (IHIFs) (providing services to their employees), and commercial health insurance organizations (providing supplementary insurance services) (Table 1). In the IHIO, four separate funds were created to ensure the coverage of the target population and financial transparency. The Imam Khomeini Relief Foundation (IKRF), as a support foundation, provided health insurance for disadvantaged families [22].

**Table 1:** Health insurance organizations (Insurers) in Iran

Category	Definition	Examples
Social Health Insurance (SHI)	SHI provides basic health insurance coverage for their beneficiaries and includes three main insurance funds	Iran Health Insurance Organization (IHIO) Social Security Organization (SSO) Armed Forces Medical Services Insurance Organization (AFMSIO)
Institutional Health Insurance Funds (IHIFs)	These funds provide health insurance coverage to their employees individually as a fringe benefit	17 IHIFs are run by the Petroleum Industry Health Organization, the National Broadcasting Organization, banks, and other organizations that provide the required insurance services to their employees
Commercial health insurance organizations	These organizations operate voluntarily and provide supplementary private insurance	Examples of such funds include Alborz, Mellat, Pasargadae, Atieh Sazane Hafez, etc

Despite the challenges in financing the health system and health insurance ecosystem of Iran, studies on the communication network of the actors in this ecosystem from the perspective of institutions and the inter-institutional and organizational partnership are limited [16]. So far, studies have mostly analyzed the stakeholders in the health system and insurance [27–33], and studies with a network analysis approach to the relationship between the actors of the health system and insurance are rare. Therefore, the present study aims to analyze the network of actors in the health insurance ecosystem before and after the Universal Health Insurance Law in Iran.

## Methods

### Study design

**Fig. 1:**
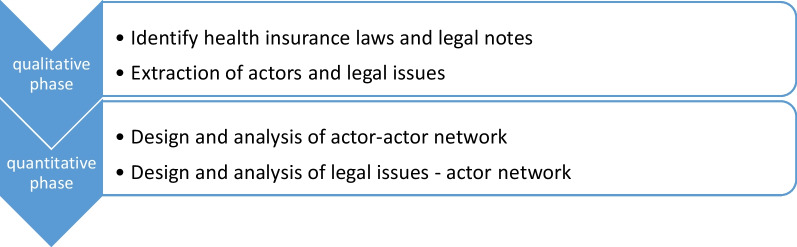
The study phases

This study has been carried out with sequential exploratory mixed method research, combining qualitative methodology using document review and directed content analysis and with quantitative methodology using the social network analysis method. The study was conducted in 2 phases (Fig. 1). First, the actors and issues related to the laws of the Iranian health insurance ecosystem from 1971 to 2021 were identified. Then, using the social network analysis method, communication networks of the actors in Iran's health insurance ecosystem were illustrated and were analyzed through network indicators.

### Qualitative data collection

For identification of the laws related to health insurance in Iran, a systematic search was conducted in the laws and regulations section of the Research Center of the Islamic Legislative Assembly [34] website in July 2021, using the keywords “Health Insurance”, “Social Security Insurance”, “Supplementary Insurance”, “Complementary Insurance”, and “Complementary Health Insurance”, from March 1971 to March 2021 (a period of 50 years). Initially, 345 laws were identified, including regulations, approvals of the Cabinet of Ministers, annual budget laws and approvals, articles of association, and determination of criteria. In searching the website, keywords had to be entered separately (using “AND” and “OR” as search commands simultaneously was not possible). Therefore, based on the search keywords, duplications of identified laws and regulations were possible. Consequently, laws searched by each keyword were saved in an Excel file and aggregated at the end. After deleting duplicate laws, 245 laws remained.

### Qualitative data analysis

A three-step process was undertaken to analyze the identified laws and to extract the pertinent actors and legal issues that are present in the health insurance ecosystem. In the first step, two members of the research team carefully studied the text of laws. Legal notes related to health insurance were extracted, where an organization or institution was explicitly obliged to perform a legal duty related to health insurance. At this stage, 510 notes, clauses, or legal articles were identified. In the second step, two members of the research team carefully reviewed all individual notes and legal materials, and the names of the mentioned institutions, organizations and interested individuals or groups were identified as actors. In the third step, the content of legal issues related to each note was extracted. The subject of each note was divided based on its thematic nature into five general categories of governance, services, information, finance, and participation and cooperation, and 30 more detailed categories.

The UHI Law (approved on October 25, 1994) is the first and most important law of the insurance system of Iran. Therefore, this study considered this law a turning point and divided the network of the insurance laws into two periods before and after the adoption of this law. The actors and legal issues were examined and compared.

### Quantitative data collection

In order to draw the communication network of the actors in Iran's health insurance ecosystem from the perspectives of the actor–actor and the legal issues–actor, the data related to the nodes and links of the networks was collected.

In the actor–actor mode, communication links between the actors in the network were identified by quantifying the associating actors in the notes using Ravar Matrix software in the form of an n–n matrix. Then, the data on nodes (actors) and links (number of companions in the notes) were entered into the Gephi software.

In the legal issues–actor network, the relationship between the actors and the legal subjects were examined, and analyzed and the identified notes was classified based on similar subjects. A matrix of the relationship between the actors and the subject of legal notes was created in the Excel software using the pivot table and activating the model data option. Then, the data on nodes (actors and legal issues) and links (number of repetitions of actors' names in each note with specified legal issues) were entered into the Gephi software.

It was necessary to compare the results of the network of actors before and after the adoption of the UHI Law. Since the names of some institutions and organizations (as actors and nodes of the network) had changed over 50 years, this study mentions the organizations and institutions identified as network actors based on their 2021 names.

### Quantitative data analysis

After that the communication networks were drawn, the network indicators were calculated and analyzed. This study conducted network analysis by calculating micro- and macro-indicators using Gephi software (the definitions of the indicators are stated in Table 2). Then, the most important network actors were identified from different aspects and considering all indicators using the combined importance index described below.

**Table 2:** Indicators used in the study

Indicator type	Indicator	Definition
Macro indicators	Node	The basic unit and constituent of a network (actors) [36]
	Links	Lines that connect two nodes, in which the links may have weight (importance, distance, etc.) in a network [36]
	Density	This index is defined as the ratio of the number of all available links to all possible links [37]
Micro indicators	Weighted degree centrality	When the links between the actors have weight, this index is obtained by multiplying the weight by the number of links that enter or exit a node [37]
	Closeness centrality	The sum of geodetic paths between a node and any other node in the network [37]
	Betweenness centrality	There are a number of other vertices that must pass through a particular node to reach their shortest [38]
	Page rank	The page rank index is calculated based on the relationship of each node in its weighted activity diagram and its measurement is calculated recursively [39]
	Clustering Coefficient	This indicator shows how the nodes are located next to their neighboring nodes [40]

In network analysis, numerous indicators are used to analyze the relationship between actors and identify essential and influential actors in the network. Some indicators are related to the whole network and are macro-indicators, such as size index (number of nodes and links) and network density, and some indicators are at the level of network nodes and are called micro-indicators, such as centrality indicators and clustering index [35].

## Calculation of the combined importance index

Each network analysis index is an answer to a question from the researcher. For example, the weighted degree centrality index is the answer to the question “which organization is the most relevant ecosystem in the communication network or which organization is associated with more organizations?” or the betweenness index answers the question “which organization is the mediating point between organizations in the ecosystem?” This study uses the combined importance index to identify the actor that involves answers to all the research questions. The combined importance index is the sum of the normalized value (a number between zero and one) of all indicators. As the index of clustering coefficient was not included in the calculation of this index for the legal issues–actor network as it was not calculated by the software.

The combined index of importance is the sum of the normalized value of weighted degree centrality, closeness centrality, betweenness centrality, page rank, and clustering coefficient. The following formula was used to normalize the values of indices [41].

$${\text{x normalized}} = {{\left( {{\text{x}} - {\text{x minimum}}} \right)} \mathord{\left/ {\vphantom {{\left( {{\text{x}} - {\text{x minimum}}} \right)} {\left( {{\text{x maximum}} - {\text{x minimum}}} \right)}}} \right. \kern-0pt} {\left( {{\text{x maximum}} - {\text{x minimum}}} \right)}}$$

## Results

**Fig. 2:**
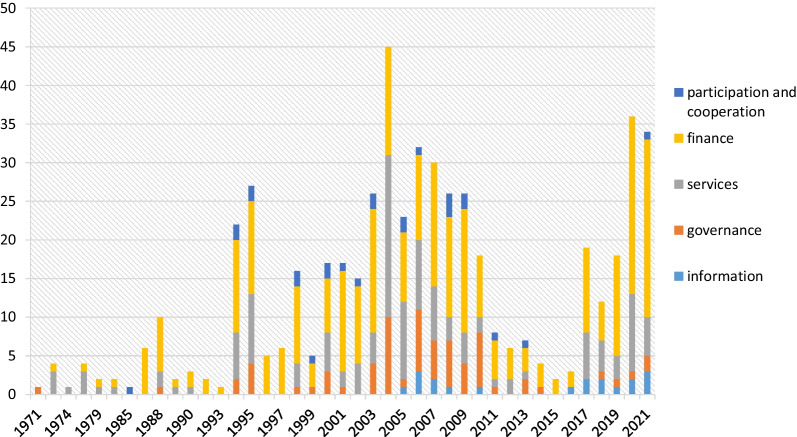
Number of legal notes in the health insurance ecosystem by type of legal subject in a period of 50 years

The present study aimed to investigate the network of the actors in the ecosystem of health insurance in Iran before and after the UHI Law in 2021. There have been 245 recognized laws in health insurance in Iran from 1971 to the end of 2021. Of these, 20 laws belong to before the UHI Law, and 225 laws have been passed afterward. The identified laws were carefully studied, and 510 notes in which an organization or institution was explicitly obliged to perform its legal duty related to health insurance were extracted. Of these, 39 belonged to the period before the UHI Law, and 471 were passed afterward. The highest number of notes were 45 notes and articles on the subject of health insurance passed in 1983. Figure 2 displays the articles in their respective categories. As can be seen, the issues of governance, information, and participation and cooperation in the insurance ecosystem have become highlighted since 1994 (after the enactment of the UHI Law). Before the enactment of the UHI Law, ecosystem laws have mostly involved services and financing, and in the whole period of 50 years, most of the legal articles and notes have been on the subject of finance.

### Legal issues–actor network before the UHI law

This network has 48 actors (organizations-legal issues) and 74 communication links between actors. The density of this network is 0.06, indicating a highly discrete communication network between organizations and insurance issues. Table 3 shows the most important legal issues of the network based on the importance index before the enactment of the UHI law. As can be seen, providing and allocating credit, receiving and paying costs, providing services, and support and supervision are ranked 1–5 as the most important issues with the highest rates in the importance index.

**Table 3:** Micro-indicators of the legal issues-actor network before the approval of the UHI law

Rank of importance index	ID node	Legal issues category	Legal issues	Weighted degree	Closeness centrality	Betweenness centrality	Page rank	Importance index
1	13	Financial	Provision, allocation of credit	0.903	1.00	1.00	1.00	1.00
2	10	Financial	Receive and pay	1.00	0.824	0.506	0.529	0.730
3	1	Services	Providing services	0.645	0.681	0.325	0.456	0.536
4	6	Services	Support services	0.226	0.639	0.336	0.435	0.414
5	9	Governmental	Monitoring	0.161	0.620	0.206	0.288	0.321
6	12	Services	Contract	0.065	0.545	0.096	0.123	0.206
7	7	Financial	Strategic buying	0.097	0.511	0.094	0.123	0.204
8	15	Services	Leveling and benefiting from services	0.161	0.545	0.017	0.099	0.204
9	2	Financial	Solvency	0.065	0.563	0.012	0.094	0.181
10	11	Participation and cooperation	Other collective structures	0.032	0.464	0.088	0.070	0.160
11	3	Services	Insurance coverage	0.065	0.341	0.093	0.144	0.157
12	5	Financial	Tariffs	0.065	0.464	0.011	0.097	0.156
13	4	Governmental	Pass a law	0.065	0.242	0.109	0.178	0.145
14	8	Services	Issuance of booklet	0.032	0.434	0.005	0.052	0.126
15	14	Financial	Premium payment	0.065	0.161	0.090	0.117	0.103

This table shows the indicators of the communication network of actors of the health insurance ecosystem with legal issues before the approval of the universal health insurance law, which are the most important legal issues ranked according to the importance index

### Legal issues-actor network after the UHI law

This network has 167 actors (organizations-legal issues) with 624 links. The network density is 0.04, meaning that only 4% out of the 100% possible connections between the actors and the laws has been established, which is 2% less than before the law. Table 4 shows the most important legal issues of the network after the adoption of the UHI Law based on the importance index. As can be seen, insurance premiums, support, tariffs, financing and allocation of credit, and commissions are ranked 1 to 5 as the most important issues with the highest rates in the importance index.

**Table 4:** Micro-indicators of the legal issues-actor network after the approval of the UHI law

Rank of importance index	ID node	Legal issues category	Legal issues	Weighted degree	Closeness centrality	Betweenness centrality	Page rank	Importance index
1	29	Financial	Premium payment	1.000	0.747	0.833	0.889	1.000
2	12	Services	Supportive services	0.489	0.767	1.000	1.000	0.938
3	11	Financial	Tariffs	0.776	0.611	0.426	0.561	0.684
4	27	Financial	Provision, allocation of credit	0.548	0.637	0.352	0.584	0.611
5	22	Participation and cooperation	Working groups	0.188	0.539	0.495	0.519	0.501
6	3	Services	Providing services	0.295	0.603	0.311	0.516	0.497
7	8	Services	Insurance coverage	0.116	0.532	0.416	0.482	0.445
8	5	Financial	Solvency	0.293	0.509	0.255	0.375	0.412
9	21	Participation and cooperation	Councils	0.241	0.488	0.199	0.336	0.363
10	26	Services	Contract	0.168	0.509	0.171	0.351	0.345
11	2	Governmental	Enforcement	0.267	0.517	0.085	0.329	0.344
12	19	Financial	Receive and pay	0.125	0.480	0.078	0.270	0.274
13	1	Governmental	Communication instructions	0.122	0.473	0.076	0.260	0.267
14	15	Governmental	Complaints handled	0.190	0.413	0.125	0.182	0.261
15	6	Financial	Franchise payment	0.145	0.439	0.057	0.204	0.243
16	7	Financial	Paying tax	0.102	0.432	0.081	0.197	0.233
17	30	Services	Leveling and benefiting from services	0.082	0.446	0.033	0.199	0.218
18	28	Financial	Determining and approving the premium	0.128	0.432	0.018	0.173	0.216
19	10	Governmental	Pass a law	0.122	0.426	0.038	0.162	0.215
20	17	Services	Issuance of booklet	0.057	0.432	0.053	0.188	0.209
21	24	Informational	Login, update and send information online	0.136	0.419	0.016	0.151	0.207
22	13	Financial	Strategic buying	0.080	0.439	0.020	0.180	0.206
23	23	Participation and cooperation	Other collective structures	0.077	0.406	0.055	0.134	0.193
24	16	Financial	Interest	0.080	0.387	0.063	0.122	0.187
25	14	Informational	System setup	0.043	0.394	0.011	0.097	0.156
26	9	Governmental	Consolidation of funds	0.020	0.369	0.059	0.087	0.153
27	25	Informational	Send offline information	0.037	0.375	0.014	0.093	0.149
28	18	Governmental	Monitoring	0.020	0.363	0.004	0.072	0.131
29	4	Informational	Notices	0.009	0.363	0.001	0.036	0.116
30	20	Informational	Eligibility based on database	0.009	0.357	0.001	0.035	0.115

This table shows the indicators of the communication network of actors of the health insurance ecosystem with legal issues after the approval of the universal health insurance law, which are the most important legal issues ranked according to the importance index

### Actor–actor network before the approval of the UHI law

This network has 33 actors (organizations) with 122 communication links between actors. The network density was 0.23, meaning that out of 100% possible connections between the network actors, only 23% was established. This indicates lower coherence between the network actors. Table 5 displays the network micro-indicators and the importance index. As can be seen, the most important actors in the network based on the importance index are the MOHME, the insured, hospitals, service centers, the SSO, and thee Planning and Budget Organization (PBO).

**Table 5:** Micro-indicators of the actor–actor network before the approval of the UHI law

Rank of importance index	ID node	Actors role	Actors	Weighted degree	Closeness centrality	Betweenness centrality	Page rank	Clustering coefficient	Importance index
1	29	Executive governance	MOHME	1.000	1.000	1.000	1.000	0.333	1.000
2	8	Insured population	Insured	0.949	0.872	0.329	0.747	0.478	0.779
3	28	Provider	Service Provider Centers	0.795	0.854	0.444	0.749	0.392	0.746
4	7	Provider	hospitals	0.718	0.854	0.471	0.716	0.467	0.745
5	21	Insurer	SSO	0.744	0.837	0.341	0.635	0.571	0.722
6	18	Financial governance	PBO	0.590	0.872	0.441	0.750	0.458	0.718
7	24	Insured population	Government employees	0.359	0.788	0.291	0.562	0.652	0.612
8	10	Provider	Physicians	0.513	0.788	0.067	0.518	0.697	0.596
9	11	Financial governance	Treasury	0.410	0.774	0.033	0.474	0.782	0.571
10	33	Legislative governance	Cabinet	0.333	0.788	0.248	0.538	0.473	0.549
11	13	Executive governance	University of Medical Sciences	0.205	0.719	0.000	0.338	1.000	0.522
12	30	Executive governance	MCLSW	0.205	0.719	0.000	0.338	1.000	0.522
13	16	Insured population	Villagers	0.179	0.683	0.021	0.306	0.857	0.472
14	9	Insurer	Basic insurer	0.154	0.631	0.000	0.261	1.000	0.472
15	5	Insured population	Vulnerable groups covered by support institutions	0.231	0.641	0.025	0.317	0.810	0.467

This table shows the indicators of the communication network of the actors of the health insurance ecosystem with each other, before the approval of the universal health insurance law, in which the most important actors are ranked according to the importance index

**Fig. 3:**
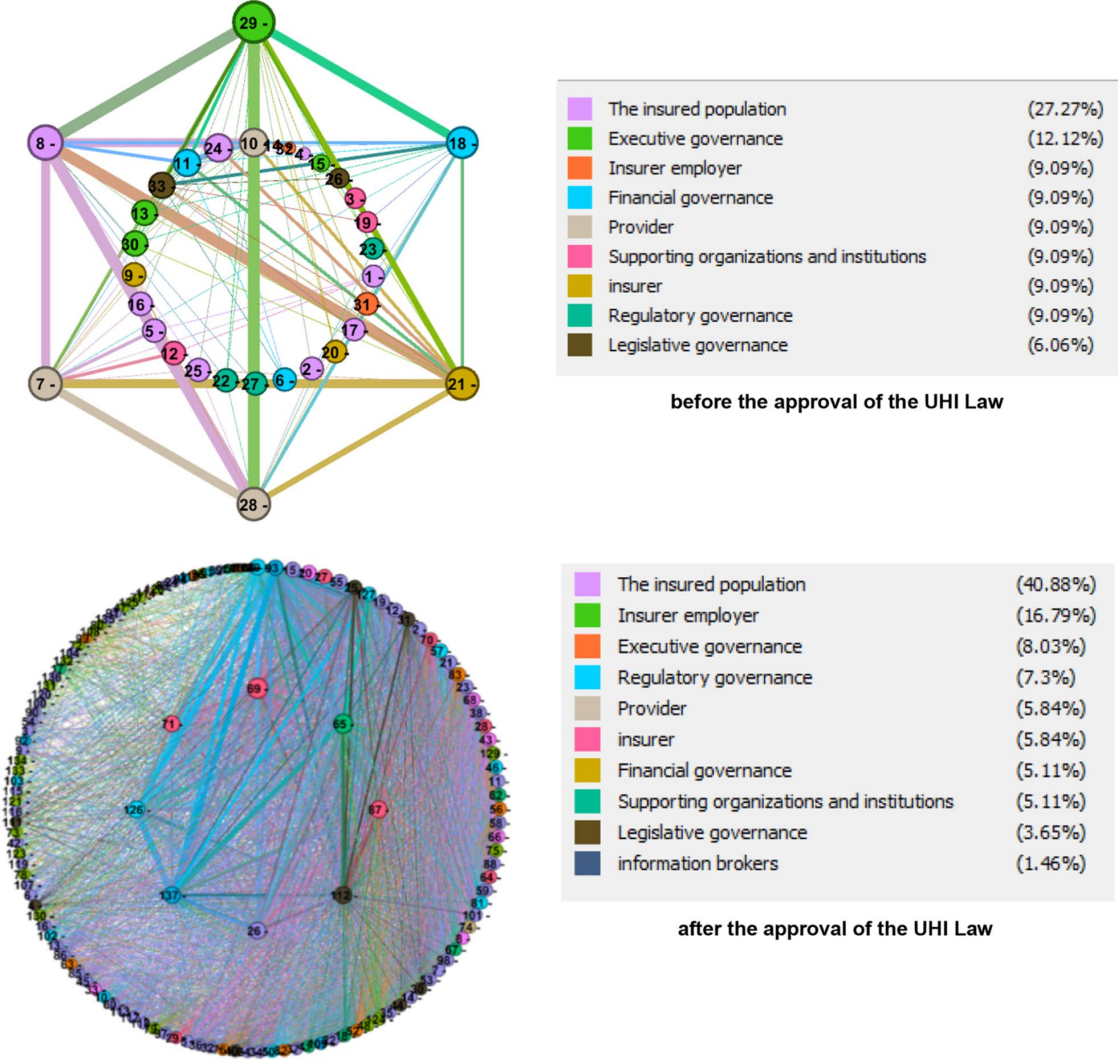
Actor–actor communication network before and after the approval of the UHI law

Figure 3 shows the communication network of the actors of the health insurance ecosystem in the context of the laws, based on their association in the notes and legal materials before the adoption of the UHI Law. Larger nodes represent the actors with a higher importance index. This means they are in contact with more actors in the network, are at the center of the communication network of the actors of the insurance ecosystem, and are closer to the center of the network. They are considered communication mediators between the actors of the insurance ecosystem, are at the receiving points of the communication flows of the insurance ecosystem, are considered more important in the course of cooperation, and are more willing to communicate with other actors. MOHME is the essential actor in this network. The thicker lines between some network actors mean that they have more connections with each other in the laws, and their relationship weighs more. For example, the lines of communication between the MOHME, the insured, service providers, and hospitals in the network are thicker than other lines, which means that these actors have more communication with each other in the context of laws.

### Actor–actor network after the adoption of the UHI law

This network has 137 actors (organizations) with 3353 communication links between actors. The network density was 0.36, meaning that out of 100% of the possible connections between the network actors, only 36% was established. This shows an increase of 0.14% than before the approval of the UHI Law, and the increase in the number of actors has created a more complex network with higher communication. Table 6 lists the names and roles of the actors and their importance index. As can be seen, the most important actor in the network based on the importance index is the IHIO, followed by the PBO, other insurance organizations, service providers, the insured, and the Cabinet. The MOHME, the SSO, the government, and the HCHI are ranked next.

**Table 6:** Micro-indicators of the actor–actor network after the approval of the UHI Law

Rank of importance index	ID node	Actors role	Actors	Weighted degree	Closeness centrality	Betweenness centrality	Page rank	Clustering coefficient	Importance index
1	69	insurer	IHIO	1.00	1.00	1.00	1.00	0.466	1.00
2	65	Financial governance	PBO	0.884	0.963	0.876	0.945	0.445	0.921
3	87	insurer	Other insurance organizations	0.794	0.939	0.493	0.843	0.491	0.797
4	112	Provider	Service Provider Centers	0.714	0.939	0.554	0.853	0.484	0.794
5	26	Insured population	Insured	0.793	0.939	0.427	0.825	0.521	0.785
6	137	Legislative governance	Cabinet	0.817	0.901	0.420	0.794	0.524	0.774
7	126	Executive governance	MOHME	0.842	0.871	0.450	0.763	0.527	0.773
8	71	insurer	SSO	0.768	0.912	0.297	0.770	0.531	0.734
9	49	Executive governance	Government	0.709	0.891	0.281	0.745	0.515	0.703
10	93	Legislative governance	HCHI	0.744	0.838	0.187	0.654	0.587	0.674
11	15	Insured population	Retirees	0.506	0.891	0.192	0.718	0.595	0.650
12	20	Supporting organizations and institutions	Martyr Foundation and Veterans Affairs	0.387	0.881	0.225	0.709	0.589	0.625
13	27	insurer	Basic insurer	0.406	0.871	0.160	0.685	0.624	0.615
14	55	Insured population	villagers	0.461	0.856	0.142	0.666	0.606	0.612
15	25	Provider	hospitals	0.430	0.816	0.245	0.642	0.551	0.601

This table shows the indicators of the communication network of the actors of the health insurance ecosystem with each other, after the approval of the universal health insurance law, in which the most important actors are ranked according to the importance index

Figure 3 shows the communication network of the actors in the context of the laws of the health insurance ecosystem created by their association in the notes and legal materials after the approval of the UHI Law. The most important node of this network, which is larger than other nodes, is IHIO. It has a higher importance index, meaning it is in contact with more actors in the network. It is closer to the center of the network, is considered a mediator and communication between the actors of the insurance ecosystem, is the receiving point of the communication flows of the insurance ecosystem, and is considered the essential actor in cooperation and more willing to communicate with other actors. The thicker lines between some network actors mean they have more communication with each other. For example, the lines of communication between the IHIO, the MOHME, the insured, service providers, the PBO, the Council of Ministers, and the HCHI are thicker than other lines in the network, meaning that their communication is heavier than the other actors.

As shown in Fig. 3, 140 actors were identified in the legal notes of the health insurance ecosystem, divided into ten roles. The number of actors has increased from 33 organizations and institutions before the law to 137 afterward. The largest share of the health insurance ecosystem before and after the enactment of the UHI Law belongs to the role of the population covered by insurance (27.27% before and 40.88% after).

## Discussion

Reforms of health system in Iran, due to the existence of many organizations with different roles and responsibilities and sometimes with similar tasks, the policy-making process is a controversial and challenging issue [12] and the implementation of any reform without the necessary scientific information can have negative effects and create conflicts and contradictions between different sectors [18]. Therefore, policy-making and reform processes require the participation of all stakeholders and actors involved to improve the system and solve complex problems [42], and the roles and responsibilities of key actors need to be clarified using network design and mapping and participatory structures strengthened [12].

The density of the actors’ network in the health insurance ecosystem has been relatively low before (0.23) and after (0.36) the enactment of the UHI Law. This indicates low coherence and correlation between actors. Studies show that in social network analysis, low network density indicates a low level of cooperation between actors. Increasing network density can strengthen weaker information links and make them formal, and increase the speed of information transfer in the network [43].

The findings of this study showed that the most important actor in the insurance ecosystem before the approval of the UHI Law is the MOHME, and after the approval of the Law is IHIO. The communication network of the actors of the health insurance ecosystem in the context of laws has become more complex after the UHI Law in 1994. Also, more actors have been added to the network, increasing their number four times that of before the law.

Studies have indicated the lack of universal and powerful governing of the communication between organizations in the Iranian health system regarding service delivery [13]. In Iran, the MOHME is the largest government agency and actor in the health insurance ecosystem network and responsible for policy-making, resource mobilization, monitoring and evaluation, and provision of health services throughout the ecosystem [26]. Before the adoption of the UHI Law, the MOHME was an actor with numerous governing roles (executive, supervisory, legislative) in the network of actors. As a result, this actor has ranked the highest in the importance index and is considered the most important network actor. The MOHME has other roles in addition to executive authority in the health insurance ecosystem, including the service provider (meaning service buyers, i.e., insurance organizations, must purchase services). Moreover, it acts as a supervisory authority over services provided by itself and other semi-public and private sectors. Experts believe one actor playing many different roles creates an environment of structural corruption [23]. The MOHME, as the administrator of the health system, should perform the role of steering, not rowing. It should create fundamental reforms to reduce its size and refine its structure and strive to eliminate conflicts of interest and corruption to achieve a desirable health status in the country [44]. In the health insurance ecosystem, interaction and cooperation between different actors and stakeholders may fail in terms of policy implementation. For example, the government may fail to provide the necessary support (particularly financial) of implementing specific policies [23].

The SSO and IHIO are the main purchasers of health services. Initially, the health insurance organization operated under the MCLSW and recently (in 2017) was transferred to the MOHME [26]. Afterward, the MCLSW lost some of its weight in the network of laws, and the Health Insurance Organization, as a fundamental insurance organization, became the most important actor in the network of the health insurance ecosystem after the UHI Law. It was expected that the SSO, as another fundamental insurance organization, would be more prominent in the context of laws along with the Health Insurance Organization, assigned more duties, and consequently, be considered an essential actor in the network. However, the results of this study proved otherwise, and the SSO was ranked eighth in the importance index.

In recent years, the progress of science and technology has introduced the role of knowledge and technology broker to this network. This role shares a comparably small part of the whole network, and it is necessary to increase its share in the entire health insurance ecosystem in the future. Knowledge and technology brokers and knowledge mediators are necessary for systems and networks to facilitate the transfer and exchange of knowledge in the face of obstacles against system reforms. Experts believe that changing the behavior of the actors can overcome these obstacles. The behavior of network actors can be changed by focusing on roles such as knowledge mediator, which facilitates interactions between actors and the transfer of knowledge within the social boundaries of the network [45]. Moreover, knowledge mediators not only create connections between different actors through the transfer of knowledge but also produce a new type of knowledge called intermediate knowledge [46].

Instead of the insurance function, the function of support services in the health insurance ecosystem became highlighted in the network so that after the approval of the UHI Law, it ranked the highest in the centrality index and page rank, and second in the importance index. Some legal issues in the law network of the health insurance ecosystem are addressed below. Support services is a legal issue that has gained importance in the network after the adoption of the UHI law. The UHI Law requires the government to provide the necessary conditions to cover all groups and individuals who apply for health insurance (with the villagers and the poor in priority). Thus, IHIO (previously Health Insurance Organization) was legally tasked with providing insurance coverage for most of the uninsured poor population and villagers. The government was obligated to undertake part of the per capita expenses of health insurance for insured villagers and secure the funds from the public budget, and dedicate a separate share in the annual budget. Also, the government was required to provide per capita insurance for medical services for disadvantaged individuals unable to pay the expenses, dedicate a part of the annual budget, and assign it to the IKRF [47].

The legal issue of paying insurance premiums has gained considerable importance after the enactment of the UHI law. Before the enactment of the law, insurance premiums ranked last in the importance index, but afterward, they reached the highest weight index in the network and, in total, first place in the importance index. Premiums are a significant source of health financing in most countries [48]. Governments can achieve sustainable financing through prepayment and premiums, as most countries approaching a comprehensive public insurance coverage and financing sustainability have done [49]. Therefore, the importance of this issue has been emphasized in the laws of the health insurance ecosystem of Iran.

Tariffing is another essential legal concept ranking high in the importance index in the network after the adoption of the UHI law. Governmental actors have power in setting medical tariffs by reviewing tariffs annually. However, no systematic, transparent process for pricing health care exists to effectively manage the conflict of interest of different actors [50]. Tariffs are an essential component of the resource allocation and purchasing process in every health system and can regulate the relationship between chief stakeholders (providers, recipients, service buyers, and payers), determine the content of the insurance package, and guide on decisions to use services in the financing system [51].

As a strength, this study provided a complete and comprehensive presentation of the communication network of the actors of the ecosystem of health insurance laws in Iran in 50 years. All actors of this network were analyzed as a comprehensive network without considering a specific legal issue (aggregation of funds, tariffs, service packages). On the other hand, Network analysis emphasizes examining and analyzing the relationship between actors throughout the whole network, rather than a specific relationship between two or more actors. In this respect, as a limitation, the results of this study should be considered from a general point of view. Since the relationship between the actors was examined in the context of the laws, they may differ from what takes place in practice in the real world.

## Conclusions

The results showed that the number of actors in the health insurance ecosystem network in the context of laws has increased after the adoption of the UHI law compared to before, while the necessary coherence and solidarity between the actors are lacking. Before UHI Law, MOHME had a significant role, but afterward, IHIO gained more importance due to its assigned responsibilities in health insurance laws, leading to the expansion of support services. As a result, sustainable financing has become challenging for the health insurance ecosystem. To overcome this challenge, dependence on government resources has increased, and the role of the PBO in the network has become more prominent. The SSO operates independently on separate principles and procedures with no regard to this law, as another fundamental insurance organization in Iran. This has created a conflict of interest between the two organizations. Legal issues surrounding premiums and tariffs on health insurance often involve various actors. To prevent conflicts of interest and improve governance, roles should be assigned clearly and independently. Introducing mediator actors in the health insurance ecosystem can strengthen governance and fill structural gaps.

## Data Availability

The data analyzed are available from the corresponding author on reasonable request.

## Abbreviations

UHI: Universal Health Insurance
IHIO: Iran Health Insurance Organization
SSO: Social Security Organization
MOHME: Ministry of Health and Medical Education
AFMSIO: Armed Forces Medical Services Insurance Organization
IKRF: Imam Khomeini Relief Foundation
HCHI: High Council of Health Insurance
MCLSW: Ministry of Cooperative Labor and Social Welfare
PBO: Planning and Budget Organization
MSIO: Medical Services Insurance Organization
IHIFs: Institutional Health Insurance Funds
WHO: World Health Organization
